# Emodin ameliorates renal injury in BXSB mice by modulating TNF-α/ICAM-1

**DOI:** 10.1042/BSR20202551

**Published:** 2020-09-18

**Authors:** Xinlu Yuan, Binbin Dai, Liyan Yang, Beiduo Lin, Enqin Lin, Yangbin Pan

**Affiliations:** 1 Department of Endocrinology and Metabolic Diseases, Shanghai Pudong Hospital, Fudan University Pudong Medical Center, Shanghai 201399, China; 2 Department of Nephrology, The First Affiliated Hospital of Fujian Medical University, Fuzhou 350005, China; 3 Department of Nephrology, Shanghai Pudong Hospital, Fudan University Pudong Medical Center, Shanghai 201399, China

**Keywords:** emodin, lupus nephritis, TNF-α, ICAM-1

## Abstract

The purpose of the present study was to explore the effects of emodin on renal injury in a BXSB mouse model of lupus and its mechanisms. BXSB mice were fed different concentrations of emodin (0, 5, 10 and 20 mg/kg.d), and the levels of intercellular adhesion molecule-1 (ICAM-1), tumor necrosis factor-α (TNF-α) and fibronectin (FN) levels in the glomeruli and serum levels of the anti-dsDNA antibody were determined. Mesangial cells (MCs) were cultured *in vitro*, and IgG-type anti-dsDNA antibody and/or emodin were added to the MC culture supernatant. In addition, TNF-α small interfering RNA (siRNA) was transfected into MCs to explore the mechanism of action of emodin. The results showed that the mice fed emodin presented decreases in the urinary protein content and glomerular TNF-α, ICAM-1 and FN levels (*P*<0.05). Moreover, the urine protein, TNF-α, ICAM-1 and FN levels were decreased in a dose-dependent manner (*P*<0.05). *In vitro*, the anti-dsDNA antibody group exhibited increased levels of ICAM-1 and TNF-α (*P*<0.05), and the anti-dsDNA antibody group showed myofibroblast-like structural changes. The aforementioned indexes were decreased in the emodin group (*P*<0.05), and the extent of transdifferentiation was significantly reduced. Moreover, the level of ICAM-1 decreased with the down-regulation of TNF-α (*P*<0.05). Emodin reduced the urine protein levels and serum levels of the anti-dsDNA antibody in a mouse model of lupus nephritis (LN). The underlying mechanism may be related to decreased levels of TNF-α, ICAM-1 and FN and the inhibition of dsDNA antibody-induced MC damage.

## Introduction

Systemic lupus erythematosus (SLE) is a multisystem autoimmune disease that commonly affects the kidneys. Lupus nephritis (LN) is the most common cause of kidney injury in patients with SLE. More than half of patients with SLE suffer from LN [1,2]. In addition, severe LN progresses to end-stage kidney disease within 15 years of diagnosis in 10–30% of patients. In fact, renal injury is the most important predictor of mortality in patients with SLE [3,4].

Currently, the treatments for patients with LN primarily consist of immunosuppressants. Standard treatments for LN remain unsatisfactory, and an accurate prediction of the responsiveness to therapy or the long-term outcome of individual patients is impossible, although a decrease in the severity of LN has recently been reported in these patients [5]. Moreover, the use of immunosuppressants may cause adverse events, such as infections [6]. Therefore, a new therapeutic strategy for LN is urgently needed.

Emodin is one of the major components of the root and rhizome of *Rheum palmatum* (Chinese rhubarb). Emodin attenuates hypoxia and LPS-induced intestinal epithelial barrier dysfunction by inhibiting the NF-κB and HIF-1α signaling pathways; it also protects against cisplatin-induced oxidative stress in cultured HEK 293 cells [7,8]. Moreover, emodin exerts a broad range of effects on the immune system. The potential immunosuppressive mechanism might be related to the suppression of lymphocyte proliferation and cytokine production, which may participate in the modulation of immune suppression and the induction of immune tolerance [9]. As shown in the study by Liu et al., emodin inhibits fibroblast proliferation and promotes programmed cell death by up-regulating the expression of the *c-MYC* gene in renal fibroblasts from subjects with LN [10]. However, the therapeutic effects and mechanisms of action of emodin in LN remain unclear.

In the present study, we investigated the therapeutic effect of emodin on LN and explored its underlying mechanisms. We expect to provide a theoretical basis for the treatment of LN with emodin and for delaying the process of LN.

## Materials and methods

### BXSB mice

Lupus-prone male BXSB mice were purchased from the Jackson Laboratory (U.S.A.). All animal experiments were conducted in the Experimental Animal Center of Fujian Medical University. The mice were bred under specific pathogen-free conditions in a room maintained at a temperature of 22°C with 60% humidity on a 12/12-h light/dark cycle and were provided free access to food and water. The present study was conducted according to the Principles of Laboratory Animal Care and approved by the Ethics Committee of Fujian Medical University (No. 056). The 24 male BXSB mice were randomly divided into four groups at the age of 3 months. Each group contained six mice and all animals had approximately the same weight (19.30 ± 0.90 g). Emodin (molecular weight = 270.23, purity 95%) was purchased from Pufei De Biotech. Co. Ltd (Chengdu, China). The four groups were fed with normal saline only (control group) or 5, 10 and 20 mg/kg.d emodin dissolved in normal saline (treatment groups) for 30 days. Mice were killed by cervical dislocation under deep isoflurane anesthesia (4% isoflurane).

### Cell culture

The mouse mesangial cell (MC) line was obtained from the Cell Resource Center, Shanghai Institutes for Biological Sciences (Shanghai, China). The cells were cultured in Dulbecco’s modified Eagle’s medium (Invitrogen; Thermo Fisher Scientific, Inc., Waltham, MA, U.S.A.) supplemented with 10% fetal bovine serum (Gibco, U.S.A.) at 37°C in an atmosphere containing 5% CO_2_. Penicillin (100 U/ml) and streptomycin (100 µg/ml) were added to the culture medium to prevent bacterial contamination. Prior to the real-time PCR, Western blot and microscopy analyses, all of the MC groups were incubated with the indicated treatments.

### Transfection

Small interfering RNAs (siRNAs) for the specific silencing of the tumor necrosis factor-α (TNF-α) gene were designed and synthesized by QIAGEN (Germany). The TNF-α siRNA was transfected according to the HiPerFect transfection reagent handbook (QIAGEN). Briefly, 3 × 10^5^ cells were seeded in six-well plates and transfected with complexes consisting of 5 nM TNF-α siRNA or a negative control scrambled siRNA. The MCs were incubated with the transfection complexes under normal growth conditions for 48 h.

### Detecting urinary protein, BUN and Scr levels

A 24-h urine sample was collected from each mouse in metabolic cages after 30 days of feeding. We adopted the quantitative method of the Pyrogallol Red protein dye-binding assay to evaluate the total protein content in the urine. The BUN and Scr levels were detected using routine biochemical methods in the clinical laboratory. The method used to obtain the sera is described below.

### Measurement of serum levels of the anti-dsDNA antibody

Serum levels of the anti-dsDNA antibody were determined using an enzyme-linked immunosorbent assay (ELISA), as previously described [11]. Briefly, microtiter plates were coated with 5 g/ml goat anti-mouse IgG (SouthernBiotech) in PBS overnight. For the detection of dsDNA-specific antibodies, the plates were coated with DNA from *Escherichia coli* (50 μg/ml in PBS, Sigma–Aldrich). Nonspecific binding was then blocked with 3% BSA in PBS, and serum samples were incubated for 1 h at 37°C. Biotinylated goat anti-mouse IgG was used as a secondary antibody, followed by an incubation with streptavidin–AP. The absorbance of the substrate was measured at 405 nm.

### Histological analysis of the renal pathology

The renal pathology was analyzed in paraffin sections of the kidney to determine the extent of renal damage and cellular infiltration. Kidneys were fixed with 4% paraformaldehyde overnight and embedded in paraffin. Sections (6 μm) were stained with Hematoxylin and Eosin (H&E) and Periodic Acid–Schiff (PAS). Glomerular, tubulointerstitial, and vascular damage were evaluated using a previously described semiquantitative scoring system [12].

### Immunohistochemical detection of intercellular adhesion molecule-1 and fibronectin expression in the glomeruli

The mice were killed and their kidneys were fixed, embedded in paraffin, and sectioned. After routine dewaxing and antigen retrieval by heating in a microwave for 15 min at a temperature > 95°C, the sections were treated with Antigen Rehabilitator I. We added the following reagents in accordance with standard immunohistochemistry (IHC) procedures. First, the diluted goat anti-intercellular adhesion molecule-1 (ICAM-1; 1:500, Abcam, U.K.) or fibronectin (FN; 1:500, Santa Cruz Biotechnology, U.S.A.) antibodies were placed on the kidney sections and incubated for 30 min at 37°C. Then, they were incubated with rabbit anti-goat-IgG-biotin (1:3000, Abcam, U.K.) and streptavidin–horseradish peroxidase (Sigma–Aldrich, America). Finally, the freshly diluted DAB-Stock Stain was used to stain the specimens for 15 min at room temperature in the dark. The specimens were lightly counterstained with Hematoxylin. Images of immunostaining in at least three separate microscopic fields of view were captured and semiquantitatively analyzed using Image-Pro Plus software (Media Cybernetics, Rockville, MD, U.S.A.) [13]. The measured parameters included the integral optical density (IOD) and sum of the area. We used the IOD of the stained area/area to represent the level of protein expression.

### Quantitative real-time RT-PCR analysis of TNF-α, ICAM-1 and FN

Total RNA was isolated from kidney samples or MCs, and first-strand cDNAs were synthesized using an oligo(dT) primer. PCR primers were designed according to the gene sequences downloaded from NCBI with the primer design software Primer 5.0. The TNF-α gene was amplified with the forward primer (5′-TGACCACCACCAAGAATT-3′) and the reverse primer (5′-TGTTCTGAAGTATTCCGATTG-3′). The ICAM-1 gene was amplified with the forward primer (5′-CTGGCAGACGAGAAGGTGGT-3′) and the reverse primer (5′-GCTCGCTCAGGGTCAGGTT-3′). The FN gene was amplified with the forward primer (5′-CCATCGCAAACCGCTGCCAT-3′) and reverse primer (5′-AACACTTCTCAGCTATGGGCTT-3′). A reference control gene (β-actin) was amplified with the forward primer (5′-AAGGAGCCCCACGAGAAAAAT-3′) and the reverse primer (5′-ACCGAACTTGCATTGATTCCAG-3′) to standardize the amounts of RNA and calculate the relative levels of TNF-α, ICAM-1 and FN expression using a GeneAmp 5700 Sequence Detection system. Realtime-PCR was performed using SYBR Green Kit (Qiagen, Hilden, Germany).

### Western blot analysis of ICAM-1 and FN levels

Protein samples were mixed with 5× lane marker sample buffer to create a 1× final solution loading buffer and then boiled for 5 min at 95°C. Next, the proteins were separated using sodium dodecyl sulfate/polyacrylamide gel electrophoresis (SDS/PAGE) and transferred to nitrocellulose membranes (GE Healthcare, U.S.A.) using a semidry blotting apparatus. An ICAM-1 rabbit polyclonal antibody (1:1000, Abcam, U.K.), an FN rabbit polyclonal antibody (1:500; Santa Cruz Biotechnology, U.S.A.), and a β-actin mouse monoclonal antibody (1:5000; Santa Cruz Biotechnology, U.S.A.) were used as primary antibodies. The Western blots were probed with a goat anti-rabbit or anti-mouse secondary antibody conjugated with IRDye 800 (1:5000, LI-COR Biotechnology, U.S.A.). Blotted proteins were detected and quantified using an Odyssey Infrared Imaging system (LI-COR Biotechnology, U.S.A.).

### Optical microscopy and electron microscopy analyses of MC transdifferentiation

Optical imaging was performed using an inverted microscope. Images were recorded with a TE-2000U bright-field optical microscope (Nikon, Tokyo, Japan). The electron microscopy (EM) analysis is described below. After treatment, the samples were prefixed with 2.5% glutaraldehyde (pH 7.4) overnight at 4°C and then postfixed with 1% osmium tetroxide for 2 h at 4°C. Then, the samples were dehydrated in a graded series of acetone solutions before endosmosis in propylene oxide for 1 h at room temperature, embedded in pure epoxy resin and polymerized at 60°C for 24 h. The samples were stained with 1% uranyl acetate for 20 min, followed by staining with 2% lead citrate for 10 min. Electron micrographs were captured using an electron microscope (EM208, Philips, Netherlands).

### Statistical analysis

All experiments were performed at least five times. The data are presented as the means ± SD. Statistically significant differences in mean values were assessed using Student’s *t* test or one-way analysis of variance (ANOVA) for multiple comparisons. Data were analyzed with SPSS version 22.0 (Armonk, U.S.A.). A value of *P*<0.05 was considered statistically significant.

## Results

### Emodin reduced the proteinuria and the level of the dsDNA antibody in the BXSB mice

We measured 24-h urinary protein excretion and the serum dsDNA antibody titer at the end of the 1-month treatment period to determine the effect of emodin on inhibiting urine protein excretion and the serum dsDNA antibody titer. Compared with the control group, the 5, 10 and 20 mg/kg.d treatment groups exhibited significantly decreased proteinuria (*P*<0.05). The serum dsDNA antibody titers were consistent with the results of proteinuria (*P*<0.05). However, the serum BUN and Scr levels were not different among the four groups (Table 1).

**Table 1:** Biochemical features of each group of BXSB mice

Group	Control	5 mg/kg.d	10 mg/kg.d	20 mg/kg.d
**Proteinuria (g/d)**	0.78 ± 0.06	0.56 ± 0.08*	0.49 ± 0.08^†^	0.38 ± 0.07^†^
**BUN (mmol/l)**	10.21 ± 2.53	9.72 ± 1.83	9.25 ± 1.80	9.10 ± 1.49
**Scr (μmol/l)**	125.45 ± 28.57	121.66 ± 21.33	119.69 ± 23.50	118.41 ± 24.52
**ds-DNA (titer)**	5.90 ± 0.62	4.85 ± 0.67*	4.29 ± 0.56*	3.74 ± 0.57^†^

* *P*<0.05 vs. control. ^†^*P*<0.01 vs. control.

### Emodin improved the renal pathology and reduced the levels of TNF-α, ICAM-1 and FN in the glomeruli

**Figure 1:**
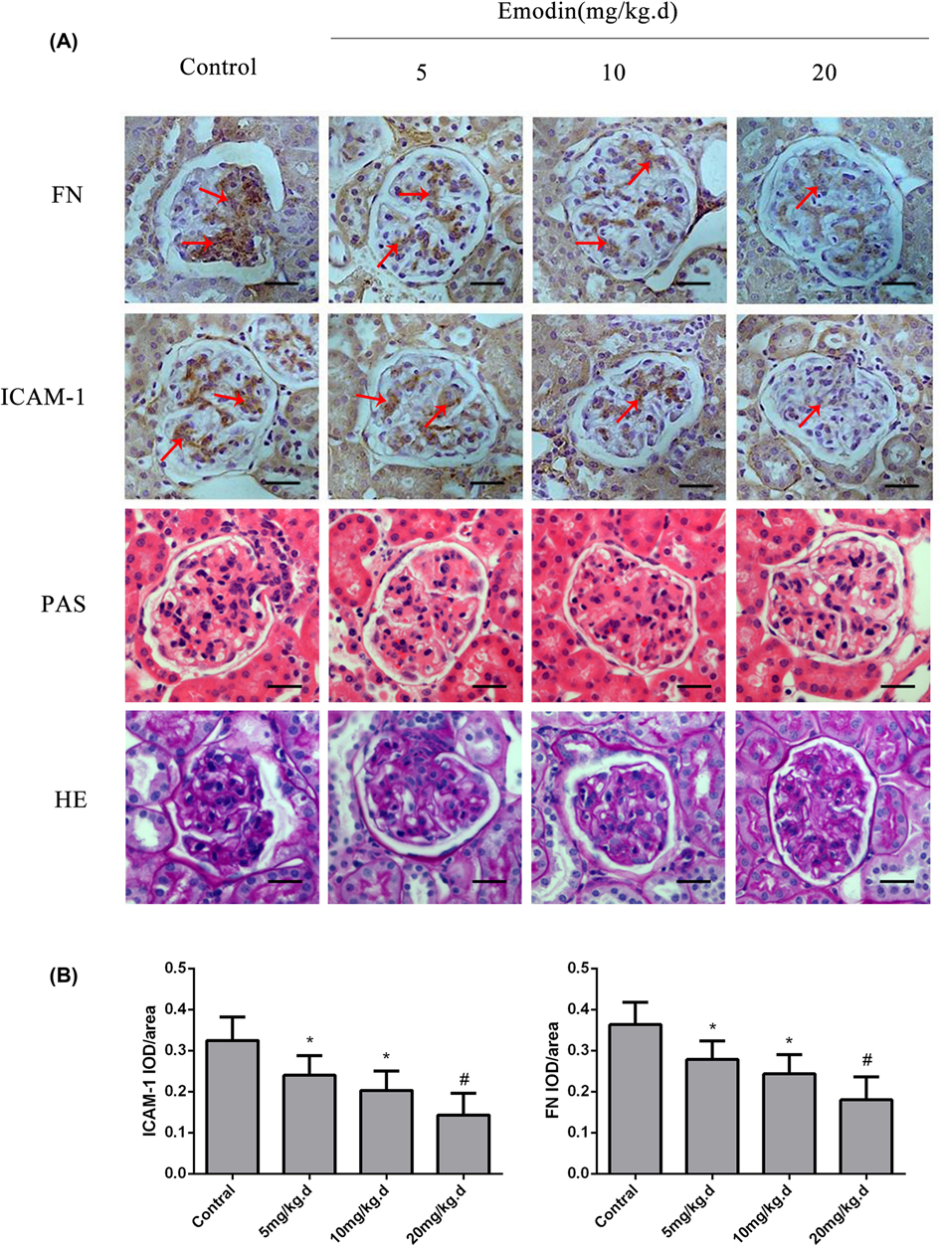
The results of IHC, H&E and PAS staining of the glomeruli of the different groups of BXSB mice (**A**) Representative images of H&E, PAS and IHC staining for ICAM-1 and FN in the kidneys of the different groups of BXSB mice (bar = 20 μm). The brown deposition indicated by the red arrow represents the proliferative level of ICAM-1 and FN in glomeruli. The darker the color, the more obvious the proliferation. (**B**) Relative expression of ICAM-1 and FN (IHC) in the glomeruli of the different groups of BXSB mice. *P<0.05 compared with the control group; ^#^*P*<0.01 compared with the control group.

**Figure 2:**
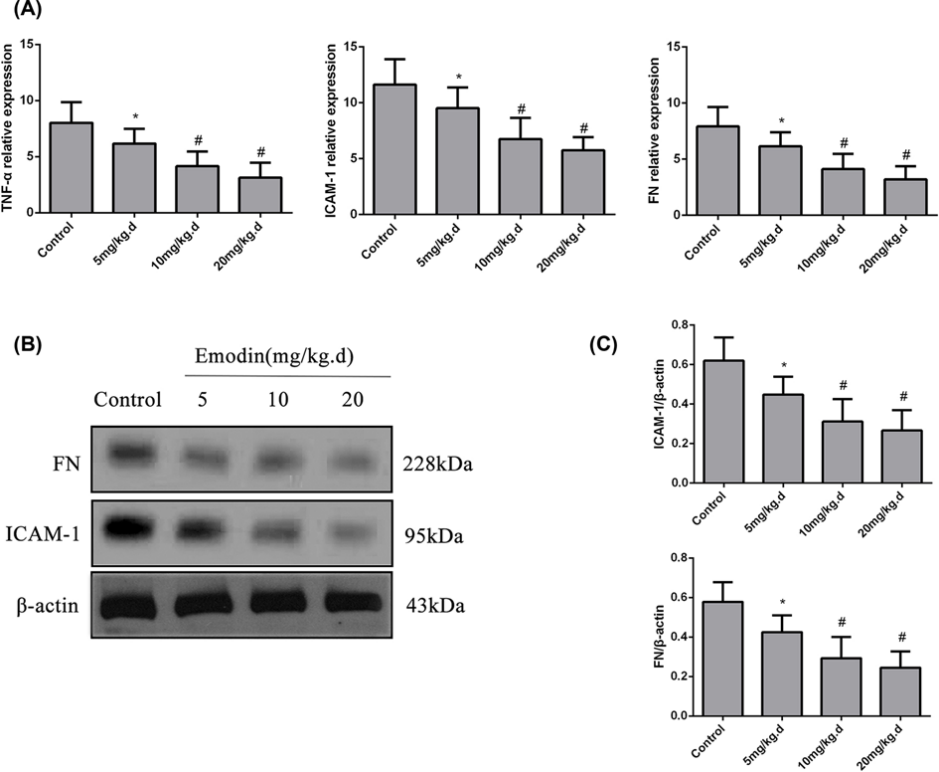
The levels of TNF-α, ICAM-1 and FN in the kidneys of the different groups of BXSB mice (**A**) The relative expression of TNF-α, ICAM-1 and FN (real-time PCR) in the kidneys of the different groups of BXSB mice. (**B**) Representative Western blots showing ICAM-1 and FN levels in the kidneys of the different groups of BXSB mice. (**C**) The relative levels of ICAM-1 and FN in the kidneys of the different groups of BXSB mice (Western blot). *P<0.05 compared with the control group; ^#^*P*<0.01 compared with the control group.

HE- and PAS-stained kidney sections were assessed by determining the histological score. The degree of the pathological changes, including mesangial cellular proliferation and mesangial matrix volume, decreased in severity as the dose of emodin increased. We also determined the renal levels of TNF-α, ICAM-1 and FN. Glomerular TNF-α, ICAM-1 and FN levels were significantly reduced in the emodin-treated mice compared with the controls (*P*<0.05). Moreover, the levels of TNF-α, ICAM-1 and FN decreased after treatment with an increasing dose of emodin (*P*<0.05, Figures 1 and 2).

### Effects of emodin on MC transdifferentiation and on the TNF-α and ICAM-1 levels *in vitro*

**Figure 3:**
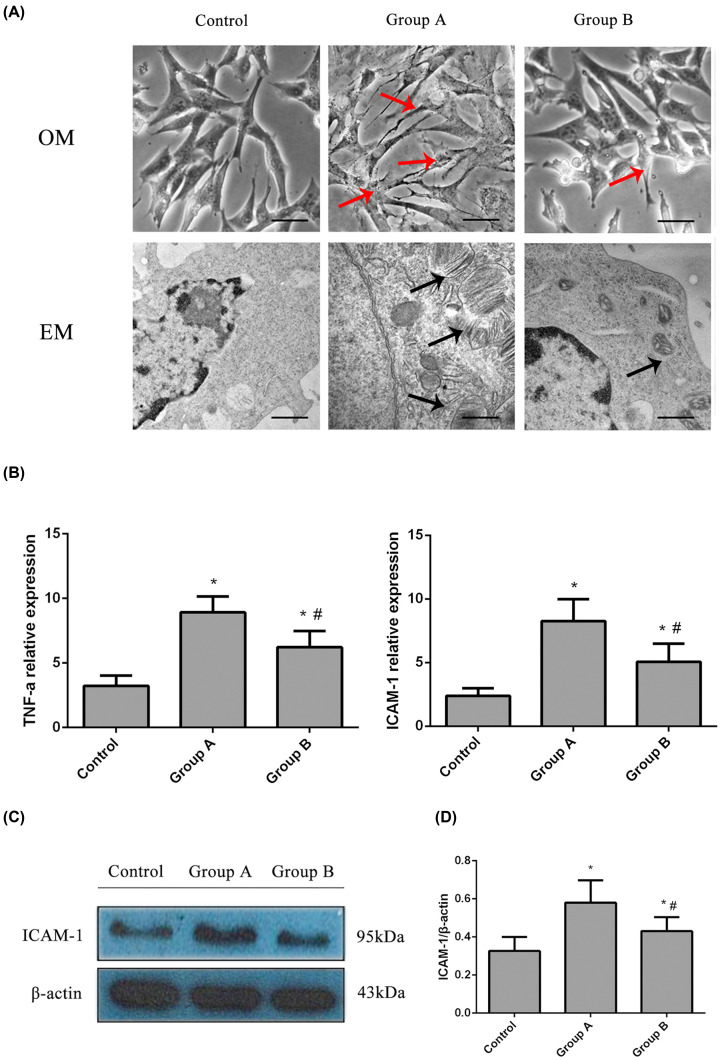
The levels of TNF-α and ICAM-1 and the ultrastructure of the different MC groups treated with or without emodin (**A**) Representative bright-field optical microscopy (OM, bar = 50 μm) and EM images (bar = 250 nm) of the transdifferentiation of MCs in the different groups. Anti-dsDNA antibodies induced myofibroblast-like phenotype transdifferentiation of MCs (indicated by the red arrows in OM and the black arrows in EM), and emodin ameliorated the transdifferentiation. (**B**) Relative expression of ICAM-1 and TNF-α in the MCs of the different groups (real-time PCR). (**C**) Representative Western blots showing the ICAM-1 and TNF-α levels in MCs from the different groups. (**D**) Relative ICAM-1 levels in MCs from the different groups (Western blot). *P<0.05 compared with the control group; ^#^*P*<0.05 compared with Group A. Control, normal group; Group A, anti-dsDNA group; Group B, anti-dsDNA + emodin group.

Cells were treated with the IgG subtype of anti-dsDNA antibodies (0.1 ml) in the presence or absence of emodin (40 μmol/l) for 72 h to determine the effects of emodin on cultured MCs. The IgG subtype of the anti-dsDNA antibodies was prepared as described in the literature [14]. Subsequently, the cells were prepared for assays. Representative bright-field optical microscopic and electron microscopic images of MC transdifferentiation in the different groups are shown in Figure 3A. Observations of the ultrastructure of the MCs showed that emodin inhibited the transdifferentiation of the MCs into the fibroblast-like phenotype. Representative gels are shown in Figure 3C. The levels of ICAM-1 and TNF-α were significantly reduced in the emodin-treated MCs compared with the control cells (*P*<0.05, Figure 3B,D).

### Effects of TNF-α down-regulation on the emodin-induced inhibition of MC transdifferentiation

**Figure 4:**
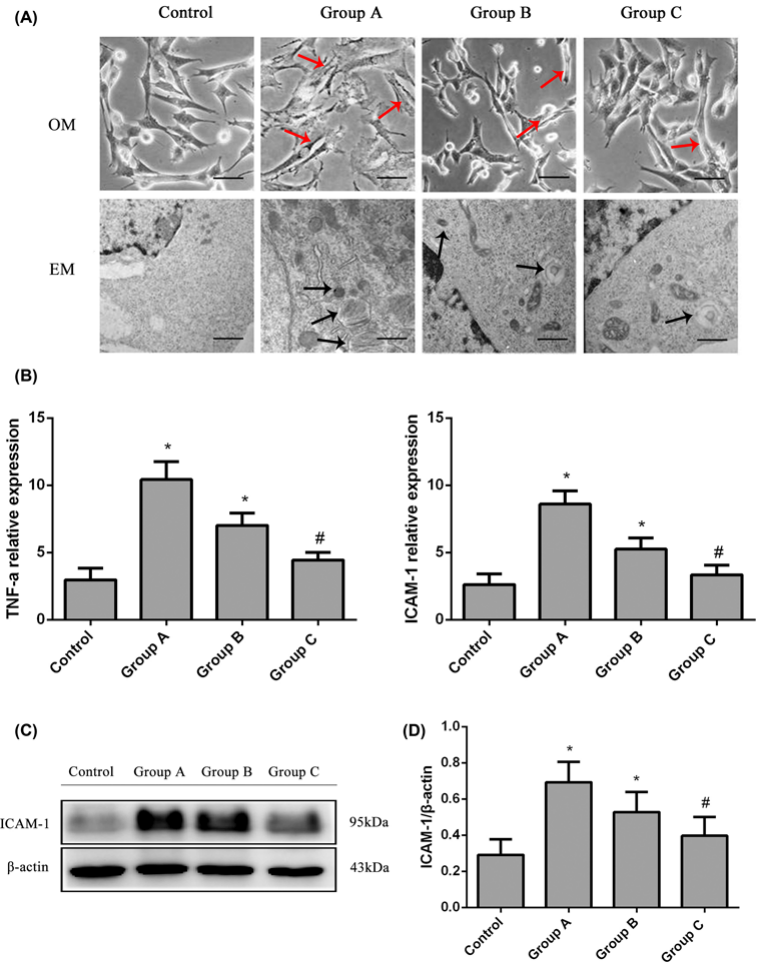
The levels of TNF-α and ICAM-1 and the ultrastructure of different MC groups treated with or without the TNF-α siRNA (**A**) Representative bright-field optical microscopy (OM, bar = 50 μm) and EM images (bar = 250 nm) of the transdifferentiation of MCs in the different groups. Anti-dsDNA antibodies induced myofibroblast-like phenotype transdifferentiation of MCs (indicated by the red arrows in OM and the black arrows in EM), and the transdifferentiation was ameliorated after TNF-α down-regulation. (**B**) Relative ICAM-1 and TNF-α expression in the MCs from the different groups (real-time PCR). (**C**) Representative Western blots showing ICAM-1 and TNF-α levels in MCs from the different groups. (**D**) Relative ICAM-1 expression in MCs from the different groups (Western blot). *P<0.05 compared with the control group; ^#^*P*<0.05 compared with Group A. Control, normal group; Group A, anti-dsDNA group; Group B, scrambled siRNA + anti-dsDNA + emodin group; Group C, TNF-α siRNA + anti-dsDNA + emodin group.

We silenced TNF-α expression in MCs by transfecting them with TNF-α siRNA to evaluate the effects of TNF-α on the emodin-induced inhibition of MC transdifferentiation. MCs transfected with the control siRNA were used as the control. Subsequently, the control and experimental cells were treated with the IgG subtype of anti-dsDNA antibodies (0.1 ml) in the presence or absence of emodin (40 μmol/l) for 72 h. Representative bright-field optical microscopic and electron microscopic images of MC transdifferentiation in the different groups are displayed in Figure 4A. Representative gels showing ICAM-1 expression are presented in Figure 4C. TNF-α expression was down-regulated by 33% in MCs transfected with the TNF-α siRNA. ICAM-1 levels were reduced in the TNF-α siRNA-transfected MCs compared with the control cells (*P*<0.05, Figure 4B,D). The MCs transfected with the TNF-α siRNA displayed lower levels of transdifferentiation.

## Discussion

In our study, the level of proteinuria decreased in BXSB mice with LN that were treated with increasing doses of emodin. Glomerular TNF-α, ICAM-1 and FN levels were significantly reduced in emodin-treated mice, and the serum dsDNA antibody titer was negatively correlated with the emodin dose. *In vitro*, ICAM-1 and TNF-α levels were significantly increased after the IgG-type anti-dsDNA antibody was added to the MC culture supernatant. Moreover, IgG-type anti-dsDNA antibodies promoted the transdifferentiation of the MCs into a myofibroblast-like phenotype. However, these changes were reduced after emodin was added.

BXSB mice are an animal model of inherited LN with defects in their autosomes [15]. The SLE-prone BXSB mice develop severe LN and produce various autoantibodies [16]. Usually, the male BXSB mice begin to suffer from typical LN when they are 2–3 months old. In the present study, although the BUN and Scr levels of the BXSB mice were not significantly different among the groups, the urinary protein and serum dsDNA antibody titers were markedly decreased in the BXSB mice treated with emodin. The histology of the glomeruli also showed favorable changes. Thus, emodin ameliorated the proteinuria in the BXSB mice. Moreover, emodin markedly decreased the expression of TNF-α, ICAM-1 and FN in the glomeruli, consistent with the changes in the urinary protein levels.

One of the prominent features of LN is myofibroblast activation or the transdifferentiation of MCs. MCs are specialized smooth muscle cells that are required to maintain the normal structure and function of the glomerulus [17]. Disorders of MCs usually cause nephritic syndrome, as represented by the presence of a large amount of proteinuria. MCs and their matrix form the central stalk of the glomerulus and interact closely with endothelial cells and podocytes [18]. In our study, the IgG-type anti-dsDNA antibody promoted the transdifferentiation of MCs into a myofibroblast-like phenotype. However, these changes were reduced by emodin treatment.

MCs actively contribute to the functional and morphological changes observed in the glomerulus during LN. Emodin has been shown to exert anti-inflammatory effects [19], and emodin also attenuates glucose-induced matrix synthesis in human peritoneal mesothelial cells [20]. Moreover, emodin attenuates inflammation by inhibiting the TLR2-mediated NF-κB signaling pathway, which may contribute to the regulatory effects of emodin on the immune system and inflammation in models of LPS-induced acute kidney injury [21]. Other researchers have reported that emodin inhibits cell proliferation and FN expression in MCs exposed to high glucose by suppressing the p38 mitogen-activated protein kinase (MAPK) pathway [22,23].

FN is an important component of the ECM, and high FN expression has been detected in the glomerular mesangial region. Higher expression of FN was reported to be involved in the progression of renal fibrosis [24], consistent with our data. In the present study, we have shown for the first time that the MC transdifferentiation triggered by IgG-type anti-dsDNA antibodies is ameliorated by emodin treatment via inhibition of the TNF-α/ICAM-1 axis in BXSB mice with LN.

ICAM-1, a cell surface glycoprotein, plays a role in regulating the interactions among immune cells [25]. Increased glomerular expression of ICAM-1 is commonly observed in patients with renal diseases, including LN [26–28]. Several experimental findings support a possible role for adhesion molecules in the development of LN. Up-regulation of ICAM-1 has been reported in the glomerular mesangium and on the endothelium in a murine model of LN [29,30]. ICAM-1 expression on mesangial and epithelial cells is induced by various cytokines [30]. This interaction with target cells is mediated through its ligand, LFA-1. The key role of ICAM-1–LFA-1 interactions in the regulation of immune reactions is also evidenced by the ability of cytokines such as TNF-α to up-regulate ICAM-1 expression [31].

TNF-α is a prominent proinflammatory cytokine. The down-regulation of TNF-α expression is considered beneficial to protect the kidneys from injuries. Anti-TNF-α antibodies have been reported to protect the kidney from ischemia–reperfusion injury [32]. Does TNF-α modulate MC transdifferentiation in individuals with LN through ICAM-1? The level of ICAM-1 was reduced in MCs transfected with TNF-α siRNA. Moreover, MCs displayed reduced transdifferentiation in response to decreased levels of TNF-α and ICAM-1.

## Conclusions

Based on our results, emodin reduced urine protein and serum anti-dsDNA antibody levels in a BXSB mouse model of lupus. The transdifferentiation of MCs to a fibroblast-like phenotype was inhibited by emodin. The immunopharmacological mechanism may be related to decreased levels of TNF-α, ICAM-1 and FN in the glomeruli and the inhibition of dsDNA antibody-induced MC damage. The present study provides a theoretical foundation for the clinical application of emodin as a treatment for LN.

## Abbreviations

BUN: blood urea nitrogen
ECM: extracellular matrix
EM: electron microscopy
FN: fibronectin
HIF-1α: hypoxia activates hypoxia-inducible factor 1
ICAM-1: intercellular adhesion molecule-1
IOD: integral optical density
LFA1: lymphocyte function associated antigen-1
LN: lupus nephritis
LPS: lipopolysaccharide
MC: mesangial cell
PAS: Periodic Acid–Schiff
Scr: serum creatinine
siRNA: small interfering RNA
SLE: systemic lupus erythematosus
TNF-α: tumor necrosis factor-α
